# Fatigue in chronically critically ill patients following intensive care - reliability and validity of the multidimensional fatigue inventory (MFI-20)

**DOI:** 10.1186/s12955-018-0862-6

**Published:** 2018-02-20

**Authors:** Gloria-Beatrice Wintermann, Jenny Rosendahl, Kerstin Weidner, Bernhard Strauß, Andreas Hinz, Katja Petrowski

**Affiliations:** 1 Department of Psychotherapy and Psychosomatic Medicine, Medizinische Fakultät Carl Gustav Carus, Technische Universität Dresden, Dresden Fetscherstraße 74, 01307 Dresden, Germany; 2 Center for Sepsis Control and Care, Jena University Hospital, Friedrich-Schiller University, Jena, Germany; 3 Institute of Psychosocial Medicine and Psychotherapy, Jena University Hospital, Friedrich-Schiller University, Jena, Germany; 4 Department of Medical Psychology and Medical Sociology, University of Leipzig, Leipzig, Germany

**Keywords:** Fatigue, Intensive care, Multidimensional fatigue inventory (MFI), Chronic critical illness (CCI), Sepsis, Health-related quality of life, Post-intensive care syndrome (PICS), Reliability, Validity, Psychometrics, Assessment

## Abstract

**Background:**

Fatigue often occurs as long-term complication in chronically critically ill (CCI) patients after prolonged intensive care treatment. The Multidimensional Fatigue Inventory (MFI-20) has been established as valid instrument to measure fatigue in a wide range of medical illnesses. Regarding the measurement of fatigue in CCI patients, the psychometric properties of the MFI-20 have not been investigated so far. Thus, the present study examines reliability and validity of the MFI-20 in CCI patients.

**Methods:**

A convenience sample of *n* = 195 patients with Critical Illness Polyneuropathy (CIP) or Myopathy (CIM) were recruited via personal contact within four weeks (t1) following the transfer from acute care ICU to post-acute ICU at a large rehabilitation hospital. *N* = 113 (median age 61.1 yrs., 72.6% men) patients were again contacted via telephone three (t2) and six (t3) months following the transfer to post-acute ICU. The MFI-20, the Euro-Quality of Life (EQ-5D-3 L) and the Structured Clinical Interview for the Diagnostic and Statistical Manual of mental disorders DSM-IV (SCID-I) were applied within this prospective cohort study.

**Results:**

The internal consistency Cronbach’s α was adequate for the MFI-total and all but the subscale Reduced Motivation (RM) (range: .50–.91). Item-to-total correlations (range: .22–.80) indicated item redundancy for the subscale RM. Confirmatory Factor analyses (CFAs) revealed poor model fit for the original 5-factor model of the MFI-20 (t2/t3, Confirmatory Fit Index, CFI = .783/ .834; Tucker-Lewis Index, TLI = .751/ .809; Root Mean Square Error of Approximation, RMSEA = .112/ .103). Among the alternative models (1-, 2-, 3-factor models), the data best fit to a 3-factor solution summarizing the highly correlated factors General −/ Physical Fatigue/ Reduced Activity (GF/ PF/ RA) (t2/ t3, CFI = .878/ .896, TLI = .846/ .869, RMSEA = .089/ .085, 90% Confidence Interval .073–.104/ .066–.104). The MFI-total score significantly correlated with the health-related quality of life (range: −.65-(−).66) and the diagnosis of major depression (range: .27–.37).

**Conclusions:**

In the present sample of CCI patients, a reliable and valid factor structure of the MFI-20 could not be ascertained. Especially the subscale RM should be revised. Since the factors GF, PF and RA cannot be separated from each other and the unclear factorial structure in the present sample of CCI patients, the MFI-20 is not recommended for use in this context.

**Trial registration:**

German Clinical Trials Registration DRKS00003386. Registered 13 December 2011, retrospectively registered.

**Electronic supplementary material:**

The online version of this article (10.1186/s12955-018-0862-6) contains supplementary material, which is available to authorized users.

## Background

Fatigue is a common long-term complication after treatment on Intensive Care Unit (ICU) and goes along with a negative impact on the patients’ health-related quality of life [1–3]. A small group of seriously ill patients needs prolonged mechanical ventilation, ongoing dependence from invasive critical care techniques and persistent monitoring of e.g. cardiopulmonary functions. These patients are at increased risk to become chronically critically ill (CCI) [4, 5]. CCI patients are often faced with initial severe acute inflammatory events that cause long-term alterations in the innate and/ or acquired immune system [6]. Consequential, damages at the myelin of the peripheral nerves may follow. The extended immobilization of CCI patients leads to adverse physiological alterations and deconditioning in multiple organ systems such as muscles, bones, joints, endocrine system. The prolonged treatment on ICU may contribute to a loss of muscle mass and maximal attainable muscle tension, increasing the risk for the development of Critical Illness Polyneuropathy (CIP) or Critical Illness Myopathy (CIM). In this context, a state of ongoing exhaustion or fatigue may occur which cannot be substantially alleviated by rest and may impair the patients’ rehabilitation process [5]. Moreover, fatigue may be accompanied by additional symptoms such as neurological, immunological, gastrointestinal, genitourinary symptoms and impaired energy balance [7, 8].

Especially CCI patients are confronted with symptoms of fatigue during their physical and cognitive rehabilitation process following the ICU treatment [3, 5]. CCI patients reported symptoms of chronic exhaustion even five years after ICU discharge [9]. Additionally, the present research showed a profound overlap between the construct of fatigue and depressive symptoms [10, 11]. Hence, health care providers should be aware of fatigue and its consequences in CCI patients.

Until now, a uniform definition or pathophysiology as well as gold standard for the assessment of fatigue do not exist. Within clinical research, self-report questionnaires such as the Multidimensional Fatigue Inventory (MFI-20) [12] are commonly used to assess the severity of fatigue symptoms (e.g. in chronic fatigue syndrome, cancer, Parkinson’s disease, craniopharyngeoma). At present, findings regarding the reliability and validity of the MFI in CCI patients are lacking. There is need for a valid instrument to measure fatigue in these patients in order to complete a patient-centered outcome set, assessing impairments following ICU discharge in these patients. Above, the valid assessment of fatigue in CCI patients allows a more meaningful evaluation of the effectiveness of interventional trials targeted on the improvement of fatigue post-discharge [13]. Following, the aim of the present study was to examine the measurement properties (internal consistency, measurement error, structural -, convergent -, discriminant validity, floor/ ceiling effects) of the MFI-20 in patients with chronic critical illness.

## Methods

### Participants and study procedures

**Fig. 1:**
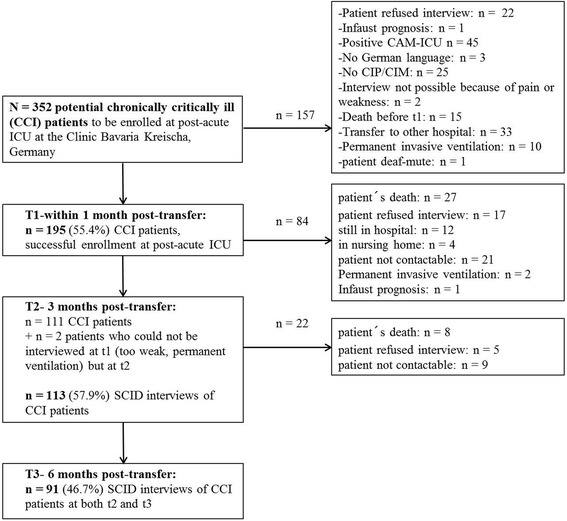
Study flow diagram. *n* = 113 patients with CCI were interviewed at t2, *n* = 91 patients with CCI were interviewed at t3. CAM-ICU: Confusion Assessment Method for the Intensive Care Unit; CIP/ CIM: Critical Illness Polyneuropathy/ Critical Illness Myopathy; SCID: Structured Clinical Interview for DSM-IV

*N* = 195 patients were consecutively enrolled in a rehabilitation hospital (Bavaria Clinic Kreischa) where they were weaned from long-term ventilation. For study participation they had to fulfill the diagnosis of a Critical Illness Polymyopathy (CIP, ICD-10: G62.80) or Critical Illness Myopathy (CIM, ICD-10: G72.80). A convenience sample of patients (see Fig. 1) with the following further inclusion criteria participated: sufficient German language skills, ICU stay of at least six days, alert and able to understand the questionnaires, transfer from ICU at acute care hospital during the weeks before inclusion in the present study. The patients were asked for study participation orally at the Bavaria Clinic Kreischa. A short cognitive test, named Confusion Assessment Method for the Intensive Care Unit (CAM-ICU) [14, 15], was applied in order to preclude cognitive impairment. The patients were informed about the study protocol and informed consent for study participation was received within four weeks following the transfer from ICU at acute care hospital to the post-acute ICU at the rehabilitation hospital (t1).

A detailed description of the study procedure has been already published elsewhere [16]. At t1, basic medical and sociodemographic data (marital status, educational level, work status, age, gender) were obtained from the patient record forms. Clinical data contained the clinical diagnoses, the severity of medical illnesses, the occurrence of sepsis, the sites of infections, the length of mechanical ventilation and the length of ICU stay. The latter two were completed at t2 and t3. The severity of the medical illnesses was assessed indirectly via the Barthel-Index (BI) [17]. The BI is a measure of performance in activities of daily living and of the early rehabilitation state (e.g. intensive care supervision, tracheostomy tube management, mechanical ventilation, confusion, severe impairment of communication, and dysphagia). It was assessed at admission and discharge from rehabilitation hospital by a trained study nurse. A minimum value of − 325 and a maximum value of 100 could be reached. Higher values indicate a better performance.

At t2 (three months post-transfer) and at t3 (six months post-transfer), the patients were contacted via telephone. During the telephone interviews, the following instruments with relevance for the present research question were applied: the MFI-20 [12], the questionnaire Euro-Quality of Life (EQ-5D-3 L) [18] and the Structured Clinical Interview for the Diagnostic and Statistical Manual of mental disorders DSM-IV (SCID-I) [19].

### Measures

The severity of fatigue was assessed with the Multidimensional Fatigue Inventory-20 (MFI-20) [12, 20] three months and six months following the transfer from acute care ICU via telephone interview. The MFI-20 is a 20-item self-report measurement of fatigue. It covers the five dimensions General Fatigue (GF), Physical Fatigue (PF), Mental Fatigue (MF), Reduced Motivation (RM) and Reduced Activity (RA). Each subscale contains two positively (e.g. „I feel very active.“) and two negatively (e.g. „I tire easily.“) formulated items. Items are rated on a 5-point Likert scale (range 1 „yes, that is true “to 5 „no, that is not true“) which are summed up to a simple total score with a minimum value of 4 (absence of fatigue) and a maximum value of 20 for each subscale. A total fatigue score is calculated as the sum of the subscale scores (range 20–100). Higher total scores indicate higher levels of fatigue. Validity has been shown for different participant populations e.g. cancer patients, army recruits, chronic fatigue syndrome. Internal consistency has been shown to be good for the GF, PF and MF dimensions (Cronbach’s α .84) [12] and adequate for the subscales RA and RM (Cronbach’s α > .65) [12].

The health-related quality of life was measured with the questionnaire Euro-Quality of Life (EQ-5D-3 L) [18] at t2 and t3. The EQ-5D-3 L assesses five dimensions (mobility, self-care, usual activities, pain/ discomfort and anxiety/ depression) which are rated within three severity levels (no problems, some or moderate problems, extreme problems or unable). A single one-dimensional index value is generated based on a simple sum score according to Hinz et al. [21]. This sum score was subjected to a linear transformation leading to values between 0 and 100. Higher values indicate a higher health-related quality of life. In the present study Cronbach’s α for the EQ-5D-3 L was .74 at t2 and .75 at t3.

The diagnosis of major depression was ascertained via SCID-I at t2 and t3. A clinical psychologist with at least five years of clinical practice applied the SCID-I via telephone contact.

The present study protocol was in consent with the Declaration of Helsinki and a positive votum was received by the Ethics Committee (No 3278–10/11) of the Friedrich-Schiller-University, Jena, Germany.

### Statistical analysis

Sociodemographic and item characteristics were displayed as frequencies. Mean values, standard deviations, medians and interquartile ranges were reported depending on the distribution of the dependent variables. Chi-squared tests or Fisher’s exact tests were applied to compare categorical data. Mann-Whitney U tests were calculated to compare ordinally or non-normally distributed data. Standardized Cronbach’s α, item-total subscale (corrected-to-total) and inter-item correlations were calculated as parameters of internal consistency. Cronbach’s α values >.70, item-total subscale (corrected-to-total) correlations ≥ .30 and inter-item correlations of .30 to .70 were acceptable [22, 23]. Measurement error, defined as systematic or random error not attributed to true changes in fatigue between the two consecutive assessments of the MFI, was calculated according to Elbers et al. [22] using Bland and Altman plots [24]. The limits of agreement were determined using the mean difference values (MFI-total, MFI subscales) between t2 and t3 ± 1.96 x standard deviation (SD) of the difference. A linear regression between difference and mean scores was applied in order to detect a proportional bias. MFI subscales were intercorrelated controlling for age and gender [12, 20, 23]. Confirmatory factor analyses (CFA) was applied using the maximum likelihood estimation (MLE) in order to assess the structural validity of the MFI-20 in the present sample of CCI patients at t2 and t3 (see Table 4, Additional file 1: Table S3). Separate CFAs were applied in the sample of CCI patients at t2 (*n* = 113) and t3 (*n* = 91). The MFI-20 may be regarded as structurally valid if the items adequately represent the proposed latent factors or measurement model. The following models were evaluated: model A assuming the original five latent fatigue factors (GF, PF, MF, RA, RM), model B assuming one single underlying latent factor, model C with a 2-factor model combining model A and B. Since the subscales representing the three factors GF, PF and RA showed high intercorrelations (see Table 3), these factors were summarized and tested in model D (3-factor model). In models A, C, and D, the latent fatigue factors were allowed to covary with mean values fixed to 0 and variances to 1. The fitness of each model with the data were evaluated using comparative fit indices (CFI, cut-off > .95; Tucker-Lewis Index, TLI,cut-off > .95) and absolute fit indices (Chi^2^ goodness-of-fit, Root Mean Square Error of Approximation, RMSEA, cut-off <.08) [25, 26]. A Chi^2^ value smaller than the number of degrees of freedom (at *p* > .05), can be regarded to be good. The CFA was carried out using IBM® SPSS® AMOS 24.0.0. The convergent validity was assessed via Spearman’s and point-bisearial correlation with the health-related quality of life and the SCID-based diagnosis of major depression. Above, the convergent validity was evaluated by composite reliability (CR > .7) and average variance extracted (AVE > .5) value of each factor. Discriminant validity was evaluated by maximum shared variance (MSV < AVE), average shared variance (ASV < AVE) and square root of AVE greater than inter-factor correlations [27]. CR, AVE, MSV, ASV and square root of AVE were calculated using stats tool package. Floor and ceiling effects were ascertained if more than 15% of the CCI patients had either the lowest or highest possible score on the MFI subscales at t2 or t3. For the statistical analyses SPSS 24.0 was used. All results were considered significant at *p* ≤. 05 (two-tailed).

## Results

Of the *N* = 352 potentially to be enrolled patients at the post-acute ICUs of the Bavaria Clinic Kreischa, *n* = 195 (55.4%) patients could be successfully interviewed within four weeks, *n* = 113 (57.9%) three months and *n* = 91 (46.7%) six months following the transfer from acute care ICU (see Fig. 1). The characteristics of the sample of CCI patients at t2 (*n* = 113) are summarized in Table 1. Non-participants and followed-up patients showed a similar age and gender distribution. More non-participants than followed-up patients had a lower degree of education. The followed-up patients showed significantly lower Barthel indices at discharge from post-acute ICU as well as from rehabilitation hospital compared with the non-participants. The mean total fatigue score was 55.9 (SD = 16.5). The mean values for the MFI subscales ranged from 8.7 for MF to 13.4 for PF. Additional file 2: Table S1 presents the medical comorbidities in both groups showing that non-participants are more often affected by specific medical comorbidities than followed-up patients. Significantly higher rates were observed for pneumonia, hypertension, organic brain syndrome and neurological disorders, by tendency only for diabetes, kidney diseases and sleep apnea.

**Table 1:** Descriptive characteristics of chronically critically ill (CCI) patients three months (t2) following discharge from ICU at acute care hospital (*n* = 113). Non-participants were defined as all patients of the potentially to be enrolled patients who could not be interviewed at t1, t2 and t3 for different reasons (see Flow chart, Fig. 1)

Characteristic	Patients *n* = 113	Non-Participants *n* = 239	U/ χ^2^	*p*
Age, yrs. median (IQR)	61.1 (55.7–65.6)	61.8 (55.5–67.1)	12,659.500	.344(U)^a^
Gender, n (%)				
Male	82 (72.6)	171 (71.5)		
Female	31 (27.4)	68 (28.5)	.039	.843(χ^2^)^b^
Family status, n (%)				
Single	10 (8.8)	29 (12.1)		
Married/cohabited	78 (69.0)	156 (65.3)		
Divorced/ living apart	16 (14.2)	31 (13.0)		
Widowed	9 (8.0)	7 (2.9) ^c^	5.476	.242(χ^2^)^b^
Education, n (%)				
< 10 yrs	35 (31.0)^d^	78 (32.6)^e^		
≥ 10 yrs	72 (63.7)	90 (37.7)	5.082	.024*(χ^2^)^b^
ICU stay, days median (IQR)	66.0 (49.0–93.5)	73.0 (52.0–115.0)	12,016.500	.095 U)
Mechanical ventilation, days median (IQR)	47.0 (33.0–70.0)	54.0 (33.0–84.0)	11,954.500	.082(U)
Sepsis, n (%)				
No sepsis	36 (31.9)	68 (28.5)		
Sepsis	42 (37.2)	96 (40.2)		
Severe sepsis or septic shock	35 (31.0)	75 (31.4)	.481	.786(χ^2^)^b^
Site of infection, n (%)				
Respiratory	56 (49.6)	125 (52.3)	.744	.689(χ^2^)^b^
Urinary/ genitals	12 (10.6)	16 (6.7)	2.061	.357(χ^2^)^b^
Abdominal	10 (8.8)	21 (8.8)	.474	.789(χ^2^)^b^
Bones/ soft tissue	6 (5.3)	12 (5.0)	.485	.784(χ^2^)^b^
Wound infection	2 (1.8)	8 (3.3)	1.176	.555(χ^2^)^b^
Heart	1 (.9)	6 (2.5)	1.525	.466(χ^2^)^b^
Multiple	13 (11.5)	22 (9.2)	.911	.634(χ^2^)^b^
Others	8 (7.1)^f^	26 (10.9)^g^	1.771	.413(χ^2^)^b^
Unknown	4 (3.5)	4 (3.5)	2.245	.325(χ^2^)^b^
Barthel index, median (IQR)				
at admission at post-acute ICU	−200.0 (− 225.0- -125.0)	−200.0 (−225.0- -125.0)	12,897.500	.490(U)^a^
at discharge from post-acute ICU	−35.0 (−82.5–7.5)	−95.0 (− 175.0- -15.0)	8475.500	<.001***(U)^a^
at discharge from rehabilitation hospital	65.0 (35.0–85.0)	−10.0 (− 150.0–60.0)	6687.000	<.001***(U)^a^
History of alcohol consumption, n (%)	22 (19.5)	43 (18.0)	.111	.739(χ^2^)^b^
History of anxiety disorder, n (%)	8 (7.1)	21 (8.8)	.296	.587(χ^2^)^b^
History of depression, n (%)	23 (20.4)	58 (24.3)	.663	.415(χ^2^)^b^
History of mental disorder, n (%)	70 (61.9)	134 (56.1)	1.089	.297(χ^2^)^b^

^a^*p*-value from Mann-Whitney-U test;

^b^*p*-value from McNemar test

^c^*n* = 16 missing values

^d^*n* = 6 missing values

^e^*n* = 71 missing values

^f^*n* = 1 brain, *n* = 5 central venous catheter, *n* = 1 port system, *n* = 1 urinary catheter

^g^*n* = 1 aorta, *n* = 1 teeth, *n* = 1 nose, *n* = 1 port system, *n* = 1 shunt, *n* = 18 central venous catheter, *n* = 1 urinary catheter, *n* = 2 heart catheter

***p ≤ .001, **p ≤ .01, *p ≤ .05

### Reliability

The internal consistency values were reasonsable for the MFI-total (Cronbach’s α = .91) and four of the five subscales (GF, PF, RA, MF, Cronbach’s α range: .69–.86). An inadequate value (Cronbach’s α = .50) was received for the subscale RM (Table 2). Regarding the inter-item correlations, a mean value of ≥ .30 was ascertained for the MFI-total and all but the RM subscale (.20, range .06–.32). The lowest correlation was present between item 15 („I have a lot of plans.“) with item 18 („I don’t feel like doing anything.”) (.06) and item 12 („I feel rested.“) (.02), suggesting item redundancy for item 15. Item-total correlations were ≥ .30 for all but the subscale RM (range: .22–.37) as well as the MFI-total (range: .23–.71) (Table 2). Pearson item subscale correlation coefficients ranged from .11 to .58 (absolute values) with the lowest and non-significant values for items 6, 11 and 20. Of them, only the removal of item 20 (“Physically I feel I am in an excellent condition.”) would lead to an increase of Cronbach’s α for the respective subscale (Additional file 3: Table S2). Values at t2 and t3 were not systematically different as shown in the Bland and Altman plots (Additional file 4: Figure S1).

**Table 2:** Item characteristics and internal consistency reliabilities for the MFI-20 subscales and total fatigue score in patients with chronic critical illness three months (t2) following the transfer from ICU at acute care hospital (*n* = 113)

	Internal consistency and reliability				
	Mean (Median)	SD (Q1-Q3)	Standardized Cronbach’s alpha^a^	Corrected-to-total correlation^a^	Inter-item correlation^a^
Total	55.9 (55.0)	16.5 (43.5–67.0)	.91	.23–.71	.35 (.02–.73)
MFI subscales					
General Fatigue (GF)	12.2 (12.0)	4.0 (9.0–15.0)	.69	.37–.56	.36 (.24–.44)
Physical Fatigue (PF)	13.4 (13.0)	3.7 (11.0–16.0)	.74	.42–.64	.42 (.28–.59)
Reduced Activity (RA)	12.6 (12.0)	4.6 (9.0–16.5)	.85	.42–.64	.42 (.28–.59)
Mental Fatigue (MF)	8.7 (8.0)	4.5 (4.0–11.0)	.86	.63–.80	.61 (.45–.73)
Reduced Motivation (RM)	8.9 (9.0)	3.5 (6.0–11.0)	.50	.22–.37	.20 (.06–.32)

^a^*N* = 113 (sample at t2); Q1-Q3 = 1st-3rd quartile

### Pairwise correlations between the MFI subscales

There was no effect of gender for all the MFI subscales. Only the MFI subscale GF was positively associated with age (Spearman’s rho = .24, *p* = .01). Nevertheless, partial correlations were calculated controlling for age and gender. At t2, the MFI subscales showed medium-sized to high correlations between each other, ranging from .37 to .77. At t3, the pairwise correlations ranged from .55 to .76 (see Table 3).

**Table 3:** Pairwise correlations between the MFI subscales at t2 and t3

	GF	PF	RA	MF	RM
	t2/t3	t2/t3	t2/t3	t2/t3	t2/t3
GF		.70^2^/.66^2^	.72^2/^.76^2^	.58^2^/.58^2^	.57^2^/.62^2^
PF	.70^1/^.67^1^		.77^2^/.75^2^	.40^2^/.51^2^	.53^2^/.63^2^
RA	.70^1/^.75^1^	.77^1/^.74^1^		.55^2^/.55 ^2^	.60^2^/.62^2^
MF	.58^1/^.57^1^	.41^1/^.50^1^	.55^1^/.51^1^		.37^2^/.64^2^
RM	.58^1/^.66^1^	.53^1/^.62^1^	.60^1^/.61^1^	.43^1^/.62^1^	

^1^Partial correlations controlling for age and gender; ^2^Spearman’s rank correlation coefficients

*GF* General Fatigue, *MF* Mental Fatigue, *PF* Physical Fatigue; *RA* Reduced Activity, *RM* Reduced Motivation

All correlations are significant at *p* ≤ .001

### Structural validity

Fit statistics revealed a poor model fit for all models at both time points (Table 4, Additional file 1: Table S3). According to the original 5-factor model (A), all but one standardized regression weights β (corresponding to factor-item correlation coefficients) were > .05 at *p* < .001. The lowest estimates were observed for item 15 for models A and B (t2/ t3, model A: β = .363/ .465, model B: β = .242/ .358). For model C, only the items of the MF subscale showed highly significant β coefficients at both t2 and t3. The best fit to the data (RMSEA ≤ .089) could be obtained for the 3-factor solution both at t2 and t3. All but items 9 and 16 were highly significantly correlated to the subscales. When each of the five factors was evaluated in separate CFAs, the factor RM turned out to show the poorest fit indices at both t2 and t3.

**Table 4:** MFI-20 models tested via Confirmatory Factor Analysis (CFA) in *n* = 113 patients three months post-ICU (t2)

Model	Number of free parameters	Chi-square (df)	*p* value	CFI	TLI	RMSEA (90% CI)
A: Original 5-Factor Model	64	401.231 (166)	<.001	.783	.751	.112 (.098–.127)
B: 1-factor model	40	503.517 (170)	<.001	.692	.656	.132 (.119–.146)
C: 2-factor model	84	345.654 (146)	<.001	.816	.760	.110 (.096–.126)
D: 3-factor model (PF/ GF/ RA summarized)	80	281.998 (150)	<.001	.878	.846	.089 (.073–.104)
Original five factors^a^						
GF	8	3.041 (2)	.219	.985	.956	.068 (.000–.212)
PF	8	.353 (2)	.838	1.000	1.050	.000 (.000–.107)
MF	8	5.491 (2)	.064	.984	.951	.125 (.000–.255)
RA	8	3.634 (2)	.163	.991	.973	.085 (.000–.224)
RM	8	8.161 (2)	.017	.769	.308	.166 (.060–.291)

*RMSEA* Root Mean Squared Error of Approximation, *CFI* Confirmatory Fit Index, *CI* Confidence Interval, *TLI* Tucker-Lewis Index, *df* degrees of freedom, *MFI-20* Multidimensional Fatigue Inventory-20, *GF* General Fatigue, *MF* Mental Fatigue, *PF* Physical Fatigue, *RA* Reduced Activity, *RM* Reduced Motivation

^a^each factor of the original MFI-20 was analyzed in independent models. In models A, C and D, the mean values of each latent variable were fixed to 0 and variances to 1. The latent fatigue factors were intercorrelated. In models B mean values and variance of the latent factor were not specified

### Convergent and discriminant validity

According to model A, the composite reliability (CR) was appropriate (CR > .7) for all MFI subscales at both time points. AVE was acceptable (> .5) besides for RM. Discriminant validity (MSV < AVE, ASV < AVE) could not be ascertained at both time points. The square root of AVE was greater than the inter-factor correlations only for the subscale MF.

According to model C, the CR was appropriate (CR > .7) only for MF at both time points. AVE was inappropriate and only acceptable (> .5) for MF at t2. Discriminant validity (MSV < AVE, ASV < AVE) could be ascertained at both time points.

Referring to model D, the CR was acceptable for all subscales besides RM. AVE was only appropriate for MF. Discriminant validity (MSV > AVE) was only appropriate for MF as well. Square root of AVE was smaller than inter-factor correlations besides for MF at t3.

Since no unequivocal factor model of the MFI-20 could be confirmed in the present sample of CCI patients, correlations were calculated only with the MFI-total but not with the single MFI subscales. The relationship between the MFI-total and the health-related quality of life (EQ-5D-3 L) yielded significant high-sized correlations at both t2 and t3 (range: − 65-(−)66). Significant small- to medium-sized correlations (range: .27–.37) could be obtained with the diagnosis of major depression (see Table 5).

**Table 5:** Convergent validity. Spearman’s correlation between the total score of the MFI-20 and EQ-5D-3 L, point-biserial correlation between the MFI-total and the diagnosis of major depression according to SCID-I

	Total Score
T2 (*n* = 113)	
EQ-5D-3 L	−.65***
Major depression (no/ yes)	.27**
T3 (*n* = 91)	
EQ-5D-3 L	−.66***
Major Depression (no/ yes)	.37***

EQ-5D-3 L = Euro-Quality of life [18]; MFI-20 = Multidimensional Fatigue Inventory [12]; SCID-I = Structured Clinical Interview for the Diagnostic and Statistical Manual of mental disorders DSM-IV [19]

***p ≤ .001; **p ≤ .01

*GF* General Fatigue, *MF* Mental Fatigue, *PF* Physical Fatigue, *RA* Reduced Activity, *RM* Reduced Motivation

### Floor and ceiling effects

There was neither a floor nor a ceiling effect for the subscales GF, PF, RA and RM. For the MF subscale, floor effects were detected. *N* = 30 patients (26.5%) had the lowest possible test score at t2, *n* = 19 patients (20.9%) at t3.

## Discussion

Fatigue is a common symptom in patients following ICU stay [13]. It is supposed that especially CCI patients are affected by a persisting feeling of exhaustion [3, 9]. However, to what extent the Multidimensional Fatigue Inventory (MFI) is a reliable and valid measure of fatigue in CCI patients, remains to be elucidated. Therefore, the present study investigated the measurement properties of the MFI in a sample of patients at three and six months following the transfer from acute care ICU to post-acute ICU at the rehabilitation hospital.

Overall, in the present sample of CCI patients the MFI values of the subscales General Fatigue (GF), Physical Fatigue (PF) and Reduced Activity (RA) were higher than those of cancer patients [28]. Likewise, CCI patients showed higher values in the above mentioned MFI subscales compared with a representative German population, whereas the subscales RM and MF were similarly scored [20]. The internal consistency was adequate for the MFI-total and the GF, PF, MF, RA subscales suggesting unidimensionality of the appropriate subscales. For the RM subscale a value smaller than the cut-off criterion of .70 for Cronbach’s α was shown. The other reliability tests (corrected item-to-total and inter-item correlation) showed inadequate values for the MFI-total and the RM subscale (values < .30). This is in accordance with the literature showing low reliability for the RM subscale either [12, 29]. One explanation might be a possible response dependency between items of this subscale [29]. Alternatively, it can be supposed that the content of the items does not correctly mirror the circumstances of the CCI patients. These often old-aged patients are in a fatal situation with a high ongoing mortality rate, increased risk for recurrent complications and persistent suffering from profound functional impairments to master basic activities of daily life. Thus, given the vague situation of CCI patients, the item content (especially from item 15: I have a lot of plans.) seems to be irrelevant for them, leading to inconsistent response patterns [12]. With respect to measurement error, the Bland and Altman plots suggest overall agreement between the assessment of fatigue at t2 and t3, with a wide range of MFI values as shown by the limits of agreement.

Floor and ceiling effects could be precluded for the MFI-total and all but the MF subscale. For the latter, 26.5% of the patient sample achieved the lowest possible test score at t2 and 20.9% at t3. This finding confirms existing results in patients with chronic illnesses (e.g. [22]) and a population-based sample of well participants [23].

In the present sample of CCI patients, a reliable factor structure of the MFI-20 could not be ascertained. Fit statistics revealed poor model fit for the original 5-factor model as originally suggested by Smets et al. [12]. Alternative models (1-factor, 2-factor) evaluated in our sample also showed no satisfying fit to the data. Fit indices rather hint towards a 3-factor solution summarizing the subscales GF/ PF/ RA. In line, other studies (e.g. [12, 22, 30]) also could not fully replicate the five factors of the MFI-20 and summarized a GF/ PF-factor. However, the model fit indices are not unequivocal since the Chi^2^ values surmount the degrees of freedom and were significant at *p* < .05. One reason for the lack of a reliable factor structure may be the high intercorrelations between the fatigue subscales as formerly shown in different patient samples (e.g. radiotherapy patients/ chronic fatigue patients/ psychology or medical students/ army recruits [12]; cancer patients [31, 32]; haemodialysis patients [26]). In the present study, the strong associations (r ≥ .70) between the subscales GF, PF and RA hint toward a large amount of shared variance. However, the 3-factor model did not reveal satisfying model fit indices as well. Regarding the high intercorrelations particularly between the subscales GF, PF, RA and the unclear factorial structure [20], the MFI-total is a more valid score for fatigue than the single MFI-subscale scores.

Consequently, a low validity of the MFI-20 has to be supposed in CCI patients. The latter has been already shown in a similar setting investigating fatigue in chronically ill haemodialysis patients [26]. Similar reasons for the lacking reliability of the factor structure can be supposed in our sample, including comprehension difficulties and a high relation of item content to the multimorbid as well as potentially life-threatening health status in the present study population. In this context, the item content of some items seems to be not relevant or inappropriate (e.g. item 6 „I think I do a lot in a day.“). In the aftermath of the ICU treatment, CCI patients are mainly involved with the recovery from the serious physical illness and their rehabilitation process [33]. The item content is therefore primarily attributed to the chronic morbidity already present in the forefront of the ICU treatment or following ICU and not to fatigue per se [26]. In line, patients particularly pay attention to the physical aspects of fatigue (e.g. vitality, activity) rather than mental complaints (e.g. concentration, mood difficulties). This is supported by significantly higher values for physical fatigue than for mental fatigue at both t2 and t3 in our CCI patients. Moreover, fatigue was predominantly related to somatic or medical reasons (e.g. efforts during rehabilitation, musculoskeletal deconditioning and general weakness following long duration of immobility, sleep disorders, pain, dyspnoea, dialysis, medication).

The models which were examined by us, only partly agreed with the criteria for convergent and discriminant validity suggested by Hair et al. [27]. Good agreement could be achieved for the subscale MF, poor agreement was obvious for RM. Above, significant correlations between fatigue and related constructs (e.g. health-related quality of life) could be shown. This has been already proven in other samples (e.g. Chronic Fatigue Syndrome, ovarian carcinoma patients) using different measurements (e.g. Short Form-36, SF-36) (see [23, 34]). Above, a significant association with depression has been shown in our sample of CCI patients and corroborates former findings where recurrent or chronic fatigue has been turned out to be a risk factor for major depression and, vice versa [35, 36]. In line, a high rate of psychiatric comorbidity has been reported in patients with chronic fatigue syndrome [11].

Our results should be interpreted in the context of the methodological strengths and limitations. The main strengths of the present study includes the investigation of a homogeneous sample of CCI patients, a prospective-longitudinal design, the long-term assessment up to six months following ICU treatment, the application of the SCID-I, and the oral data assessment via direct interviewing. The latter ensured that no missing values were produced. However, the present results should be cautiously evaluated. The kind of medical interventions or rehabilitation programmes (e.g. physiotherapy, neuropsychological interventions, ergotherapy) taking place between the assessment time points (t2 and t3) were not controlled for and might have compromised the comparability of fatigue assessment. Likewise, in the present study the time period between the assessment points was too long (three months) in order to gain an appropriate estimate for the reproducibility of the fatigue measurement over time or to calculate test-retest reliability. Although three months represents a commonly accepted interval for measuring the rate of stress-related disorders following ICU [16], future studies should apply a much shorter time frame e.g. between three and seven days when the test-retest reliability of patient-reported outcome measurements is of interest (for a systematic review see [37]). Information about the pre-ICU severity of fatigue could not be obtained, therefore the severity of fatigue found in the present study cannot be attributed to the ICU treatment as causative factor. Moreover, the rate of non-participants, although similar to other studies on patient samples after long-term mechanical ventilation (e.g. [3, 38, 39]), was quite high. The sample size of CCI patients was small. Therefore, conclusions based on the present sample should be drawn with utmost caution. Future studies are needed to replicate the results of this present study in a larger sample of CCI patients, additionally using other fatigue measures such as the Chalder Fatigue Questionnaire in order to ascertain convergent validity. Furthermore, the diagnosis of major depression should be assessed using DSM-V criteria [40]. Above, future studies should also address the minimal clinically important differences of the MFI-20 and its subscales in CCI patients (for a systematic review see [41]_ENREF_[35]).

## Conclusion

To conclude, the present study shows that the MFI-20 cannot be regarded as valid instrument for use in clinical practice and research, measuring fatigue as multidimensional construct following ICU treatment in CCI patients. Particularly the subscale Reduced Motivation showed insufficient reliability as well as validity and should be interpreted with caution. The factorial structure of the original MFI-20 could not be unequivocally approved. Our data rather hint towards a 3-factor factor solution combining General -, Physical Fatigue and Reduced Activity. Because of the unclear factorial structure, the MFI-20 cannot be considered as appropriate tool for the assessment of fatigue in CCI patients following ICU treatment. The present results demand replication in a larger sample of CCI patients. Future research according to clinical important changes of fatigue symptoms in these patients are needed using anchor-based responsiveness. Above, future research should investigate the appropriateness of the MFI-sum score in CCI patients using Rasch analysis.

## Additional files

Additional file 1: **Table S3.** MFI-20 models tested via Confirmatory Factor Analysis (CFA) in *n* = 91 patients six months post-ICU (t3). (DOCX 16 kb)

Additional file 2: **Table S1.** Medical comorbidities of the patients being followed-up (*n* = 113) and the non-participants (*n* = 239). Patients who could not be included or followed-up for different reasons were referred to as non-participants. (DOCX 17 kb)

Additional file 3: **Table S2.** Correlation coefficients of the MFI-20 items with the subscales. The item-subscale correlation coefficients are significant with **p < .01 or ***p < .001. (DOCX 15 kb)

Additional file 4: **Figure S1.** Bland and Altman plot comparing the five subscales and the total score of the MFI-20 between t2 (three months post-ICU) and t3 (six months post-ICU). Bold lines represent the mean differences, dotted lines represent the 95% limits of agreement (see Additional files). Differences and mean values were created using MFI-20 scores of t2 and t3. (JPEG 2516 kb)

## Abbreviations

AGFI: Adjusted Goodness of Fit Index
ASV: Average Shared Variance
AVE: Average Variance Extracted
BI: Barthel-Index
CAM-ICU: Cognitive Assessment for the Intensive Care Unit
CCI: Chronic Critical Illness
CFA: Confirmatroy Factory Analysis
CIM: Critical Illness Myopathy
CIP: Critical Illness Polyneuropathy
CR: Composite Reliability
DSM: Diagnostic and Statistical Manual of mental disorders
EQ-5D-3 L: Euro-Quality of Life;
GF: General Fatigue
GFI: Goodness of Fit Index
ICU: Intensive Care Unit
MF: Mental Fatigue
MFI: Multidimensional Fatigue Inventory
MSV: Maximum Shared Variance
PCA: Principal Component Analysis
PF: Physical Fatigue
RA: Reduced Activity
RM: Reduced Motivation
SCID-I: Structured Clinical Interview for the DSM-IV
SF: Short Form
