# Supplementary material for: Fatigue in chronically critically ill patients following intensive care - reliability and validity of the multidimensional fatigue inventory (MFI-20)
Source: Health Qual Life Outcomes. 2018 Feb 20;16:37. doi: 10.1186/s12955-018-0862-6 (PMC5819670; doi:10.1186/s12955-018-0862-6)
Supplement: Supplementary file 1 — Table S3. MFI-20 models tested via Confirmatory Factor Analysis (CFA) in n = 91 patients six months post-ICU (t3). (DOCX 16 kb) [file 12955_2018_862_MOESM1_ESM.docx]

**Table S3:** MFI-20 models tested via Confirmatory Factor Analysis (CFA) in n=91 patients six months post-ICU (t3).

| **Model** | **Number of free parameters** | **Chi-square (df)** | **P value** | **CFI** | **TLI** | **RMSEA (90% CI)** |
| --- | --- | --- | --- | --- | --- | --- |
| **A:** Original 5-Factor Model | 64 | 324.400 (166) | <.001 | .834 | .809 | .103 (.086-.120) |
| **B:** 1-factor model | 40 | 396.754 (170) | <.001 | .762 | .734 | .122 (.106-.137) |
| **C:** 2-factor model | 84 | 282.786 (146) | <.001 | .856 | .813 | .102 (.084-.120) |
| **D:** 3-factor model (PF/ GF/ RA summarized) | 80 | 248.492 (150) | <.001 | .896 | .869 | .085 (.066-.104) |
| **Original five factors^#^** |  |  |  |  |  |  |
| GF | 8 | 4.351 (2) | .114 | .974 | .922 | .114 (.000-.264) |
| PF | 8 | 4.224 (2) | .121 | .982 | .945 | .111 (.000-.262) |
| MF | 8 | 1.948 (2) | .378 | 1.000 | 1.001 | .000 (.000-.207) |
| RA | 8 | 1.830 (2) | .401 | 1.000 | 1.005 | .000 (.000-.203) |
| RM | 8 | 4.586 (2) | .101 | .928 | .784 | .120 (.000-.269) |

RMSEA=root mean squared error of approximation; CFI=confirmatory fit index; CI=Confidence Interval; TLI=Tucker-Lewis index; Df=degrees of freedom; MFI-20, Multidimensional Fatigue Inventory-20. GF = General Fatigue, MF = Mental Fatigue, PF = Physical Fatigue; RA = Reduced Activity, RM = Reduced Motivation; # each factor of the original MFI-20 was analyzed in independent models. In models A, C and D, the mean values of each latent variable were fixed to 0 and variances to 1. The latent fatigue factors were intercorrelated. In models B mean values and variance of the latent factor were not specified.
