# Supplementary material for: Fatigue in chronically critically ill patients following intensive care - reliability and validity of the multidimensional fatigue inventory (MFI-20)
Source: Health Qual Life Outcomes. 2018 Feb 20;16:37. doi: 10.1186/s12955-018-0862-6 (PMC5819670; doi:10.1186/s12955-018-0862-6)
Supplement: Supplementary file 2 — Table S1. Medical comorbidities of the patients being followed-up (n = 113) and the non-participants (n = 239). Patients who could not be included or followed-up for different reasons were referred to as non-participants. (DOCX 17 kb) [file 12955_2018_862_MOESM2_ESM.docx]

**Table S1:** Medical comorbidities of the patients being followed-up (n = 113) and the non-participants (n = 239). Patients who could not be included or followed-up for different reasons were referred to as non-participants.

| **Characteristic** | **Patients followed-up**  **n = 113** | **Non-participants**  **n = 239** | **χ²** | ***P*** |
| --- | --- | --- | --- | --- |
| **Medical comorbidity** |  |  |  |  |
| **Lung** |  |  |  |  |
| Chronic obstructive pulmonary disease (COPD) (J44.X) | 31 (27.4) | 80 (33.5) | 1.296 | .255 (χ²)^a^ |
| Acute respiratory insuffiency (J96.00, J96.01, J96.09) | 87 (77.0) | 198 (82.8) | 1.706 | .191(χ²)^a^ |
| Chronic respiratory insufficiency (J96.10,  J96.11, J96.19) | 10 (8.8) | 16 (6.7) | .521 | .470 (χ²)^a^ |
| Pneumonia (J15,J18,J69) | 20 (17.7) | 75 (31.4) | 7.289 | **.007**(**χ²)^a^ |
| Sleep apnea (G47.3) | 14 (12.4) | 15 (6.3) | 3.793 | .051 (χ²)^a^ |
| **Diseases of the circulatory/ cardiovascular system** |  |  |  |  |
| Left heart failure (I50.1) | 40 (35.4) | 84 (35.1) | .002 | .963 (χ²)^a^ |
| Atrial fibrillation (I48.0-I48.2) | 39 (34.5) | 79 (33.1) | .073 | .787 (χ²)^a^ |
| Hypertension (I10.0, I10.01) | 14 (12.4) | 52 (21.8) | 4.420 | **.036*** (χ²)^a^ |
| Coronary heart disease (I25.1) | 30 (26.5) | 73 (30.5) | .592 | .442 (χ²)^a^ |
| **Kidney** |  |  |  |  |
| Chronic kidney disease (N18.X) | 28 (24.8) | 84 (35.1) | 3.801 | .051 (χ²)^a^ |
| Urinary tract infection (N39.0) | 24 (21.2) | 74 (31.0) | 3.611 | .057 (χ²)^a^ |
| **Other** |  |  |  |  |
| Diabetes (E11.90) | 43 (38.1) | 67 (28.0) | 3.585 | .058 (χ²)^a^ |
| Adipositas (E66.X) | 28 (24.8) | 41 (17.2) | 2.830 | .093 (χ²)^a^ |
| Enzephalopathy (G93.4) | 27 (23.9) | 73 (30.5) | 1.668 | .196 (χ²)^a^ |
| Organic brain syndrome (F06.9) | 44 (38.9) | 127 (53.1) | 6.193 | **.013*** (χ²)^a^ |
| Neurological disorders | 27 (23.9) | 90 (37.7) | 6.549 | **.010*** (χ²)^a^ |
| Cirrhosis of the liver | 2 (1.8) | 12 (5.0) | 2.123 | .241 (†)^b^ |
| Hypothyroidism | 22 (19.5) | 59 (24.7) | 1.179 | .278 (χ²)^a^ |
| **Mental disorders** |  |  |  |  |
| History of depressive disorders | 22 (19.5) | 42 (17.6) | .185 | .667 (χ²)^a^ |
| History of anxiety disorders | 8 (7.1) | 21 (8.8) | .296 | .587 (χ²)^a^ |
| History of harmful alcohol consumption | 22 (19.5) | 43 (18.0) | .111 | .739 (χ²)^a^ |

^a^*p*-value from χ²-test; ^b^*p*-value from Fisher´s exact test
