# Supplementary material for: Fatigue in chronically critically ill patients following intensive care - reliability and validity of the multidimensional fatigue inventory (MFI-20)
Source: Health Qual Life Outcomes. 2018 Feb 20;16:37. doi: 10.1186/s12955-018-0862-6 (PMC5819670; doi:10.1186/s12955-018-0862-6)
Supplement: Supplementary file 3 — Table S2. Correlation coefficients of the MFI-20 items with the subscales. The item-subscale correlation coefficients are significant with **p < .01 or ***p < .001. (DOCX 15 kb) [file 12955_2018_862_MOESM3_ESM.docx]

| **Item** | **GF** | **PF** | **RA** | **MF** | **RM** | **Cronbach´s alpha if Item Deleted** |
| --- | --- | --- | --- | --- | --- | --- |
| 1 | **.45***** | .04 | .09 | -.09 | -.06 | .69 |
| 5 | **-.51***** | -.26 | -.14 | -.15 | -.18 | .62 |
| 12 | **.25**** | .05 | .10 | -.06 | -.11 | .58 |
| 16 | **-.52***** | -.26 | -.345 | -.35 | -.25 | .63 |
| 2 | -.17 | **-.57***** | -.10 | -.16 | -.09 | .71 |
| 8 | .25 | **.42***** | .06 | -.10 | .06 | .75 |
| 14 | -.01 | **-.40***** | -.02 | -.27 | -.18 | .62 |
| 20 | .14 | **.15** | .19 | .02 | -.06 | .65 |
| 3 | .20 | -.07 | **.29**** | -.04 | -.09 | .84 |
| 6 | -.11 | -.11 | **.12** | -.08 | -.01 | .79 |
| 10 | -.17 | -.18 | **-.51**** | -.24 | -.27 | .81 |
| 17 | -.28 | -.29 | **-.44***** | -.27 | -.22 | .79 |
| 7 | -.04 | -.08 | .01 | **.33***** | -.12 | .85 |
| 11 | -.09 | -.03 | .00 | **.11** | -.03 | .78 |
| 13 | -.18 | -.22 | -.23 | **-.38***** | -.32 | .80 |
| 19 | -.22 | -.18 | -.15 | **-.56***** | -.22 | .85 |
| 4 | .13 | -.01 | .13 | -.03 | **.45***** | .36 |
| 9 | -.09 | -.12 | -.31 | -.30 | **-.53***** | .44 |
| 15 | -.04 | -.01 | .04 | .06 | **.58***** | .50 |
| 18 | -.18 | -.30 | -.15 | -.32 | **-.47***** | .422 |

**Table S2:** Correlation coefficients s of the MFI-20 items with the subscales. The item-subscale correlation coefficients are significant with **p < .01 or ***p < .001.

GF = General Fatigue, MF = Mental Fatigue, PF = Physical Fatigue; RA = Reduced Activity, RM = Reduced Motivation
