# Supplementary figures and images for: Fatigue in chronically critically ill patients following intensive care - reliability and validity of the multidimensional fatigue inventory (MFI-20)
Source: Health Qual Life Outcomes. 2018 Feb 20;16:37. doi: 10.1186/s12955-018-0862-6 (PMC5819670; doi:10.1186/s12955-018-0862-6)

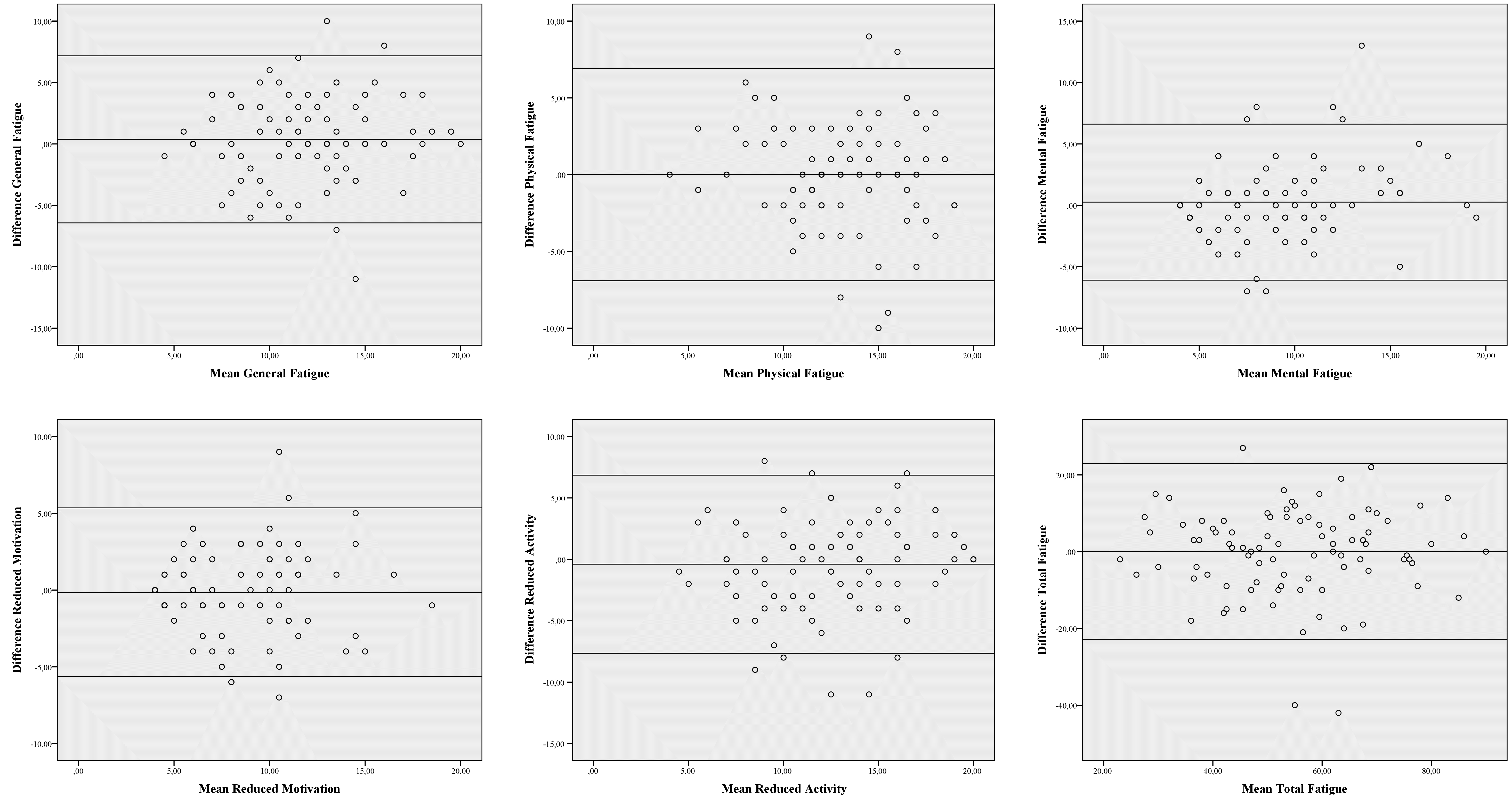

Supplement: Supplementary file 4 — Figure S1. Bland and Altman plot comparing the five subscales and the total score of the MFI-20 between t2 (three months post-ICU) and t3 (six months post-ICU). Bold lines represent the mean differences, dotted lines represent the 95% limits of agreement (see Additional files). Differences and mean values were created using MFI-20 scores of t2 and t3. (JPEG 2516 kb) [file 12955_2018_862_MOESM4_ESM.jpg]
